# Case Report: Diagnosis of hypogeusia after oral exposure to commercial cleaning agent and considerations for clinical taste testing

**DOI:** 10.12688/f1000research.11241.2

**Published:** 2017-06-19

**Authors:** Marie Jetté, Catherine Anderson, Vijay Ramakrishnan

**Affiliations:** 1 Department of Otolaryngology, University of Colorado School of Medicine, Aurora, CO, 80045, USA

**Keywords:** dysgeusia, hypogeusia, taste, tongue, burning mouth syndrome

## Abstract

Few reports in the literature document acute taste disturbance following exposure to toxic chemicals. We describe the case of a 54-year-old man who presented with primary complaint of tongue numbness and persistent problems with taste 1.5 years following oral exposure to a commercial cleaning agent. A test of olfaction revealed normosmia for age and gender. Lingual tactile two-point discrimination testing showed reduced somatosensation. Taste threshold testing using a 3-drop method demonstrated severe hypogeusia, though the patient was able to discriminate tastants at lower concentrations with a whole mouth swish and spit test. We conclude that clinical evaluation of dysgeusia can be performed using a number of previously published testing methods, however, determining causative factors may be confounded by duration since exposure, lack of knowledge of baseline taste function, and medications. Although many testing options exist, basic taste testing can be performed with minimal expertise or specialized equipment, depending on the patient history and goals of evaluation.

## Introduction

Disordered taste, referred to as dysgeusia, can lead to dramatic changes in weight and reduce quality of life. Causes for dysgeusia are variable and include primary medical ^1^ and psychological disorders ^2^, medication side effects ^3^, chemicals and toxins ^4^, local disorders of the mouth ^5^, insufficient production of saliva ^6^, surgery ^7, 8^, and gastroesophageal reflux disease. Here we describe a case of hypogeusia following oral contact with a commercial cleaning agent, and discuss diagnostic considerations in determining the nature and extent of taste dysfunction.

## Patient information

A 54-year-old Caucasian man presented with complaints of a dull sensation of the tongue, describing that his “taste seems off,” associated with numbness and occasional dry mouth, but not with flavor perception. The patient reported that the taste disturbance began 1.5 years prior to presentation, immediately after accidental oral exposure to a cleaning agent, which he gargled with and immediately expectorated. At the time of injury he noted tongue numbness and throat irritation, and subsequently reported to the emergency department. There, examination of the oral cavity revealed no findings indicative of serious mucosal injury such as significant swelling, deep ulceration, or chemical burn. At an urgent dental visit one day following the incident, the patient reported tongue burning and tingling sensations, with increased tooth and gum sensitivity, and the dentist’s examination noted superficial irritation on the tongue and cheeks along with pharyngeal erythema. The patient was seen by an otolaryngologist approximately 3 weeks following the incident for complaints of burning and soreness of the tongue and loss of taste. His physical exam was notable for an area of mild erythema of the right lateral tongue, tender to palpation but without ulceration, and he was prescribed a supersaturated calcium phosphate rinse for oral mucositis and topical benzocaine for symptomatic relief. Approximately 6 weeks following the incident, the patient was seen in follow-up by the otolaryngologist for tongue soreness. At that time, examination revealed erythema and shallow erosion/ulceration of the dorsal tongue just anterior to the foramen cecum, and light erythema of the lateral tongue. The patient was diagnosed with candidiasis and prescribed nystatin. A follow-up appointment approximately 9 weeks after the incident showed resolution of physical exam findings, however, he continued to have symptoms.

**Figure 1.**
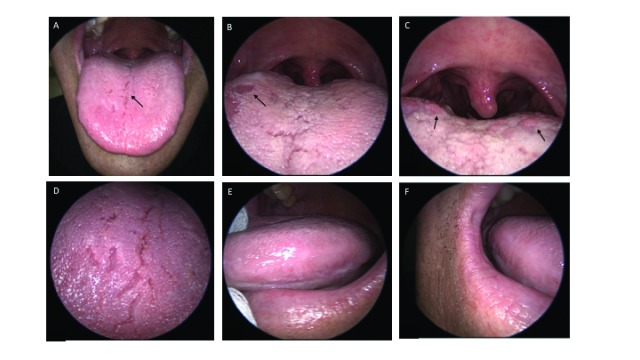
Images of patient’s tongue taken 1.5 years following oral exposure to toxic cleaning agent. The oral mucosa appears normal, though there is some evidence of possible geographic tongue. Panels **1A** and **1D** show a slight fissure (arrow). Panel **1B** demonstrates an atrophic patch (arrow) consistent with possible geographic tongue. The circumvallate papillae are intact as demonstrated in **1C** (arrows). The lateral lingual mucosa appears normal ( **1E**, **1F**).

The patient presented to our department 1.5 years after the injury, for evaluation of persistent subtotal loss of tongue sensation, both taste and somatosensation. During this visit, a complete head and neck examination was performed and was essentially normal, specifically with normal ear examination and intact cranial nerve exam. Intraoral examination demonstrated normal mucosa with no evidence of erythema or ulceration ( Figures 1C, 1E, 1F). A shallow fissure was noted along the medial surface of the tongue ( Figures 1A, 1D). An atrophic patch was noted on the posterior right tongue ( Figure 1B), possibly consistent with geographic tongue. The patient’s medical history was significant for hypertension and his surgical history was unremarkable. His body mass index was 25.8, and had not changed since the injury. Medications at the time of our evaluation included aspirin 81mg, lisinopril, as needed naproxen for musculoskeletal pains, and various vitamins and supplements including zinc.

A series of tests was assembled and administered to determine the nature and extent of the patient’s dysgeusia, including: two-cup forced choice test, two-point tactile discrimination, trigeminal nerve testing, three-drop taste threshold testing, and swish and spit whole mouth taste testing ( Table 1).

**Table 1.** Taste test battery used to assess patient.

Test	Objective	Supplies	Completion time
**Smell** **Identification** **Test (SIT)**	Olfactory function	Commercial test book	10–15 min
**Two-Point** **Tactile** **Discrimination**	Somatosensory function	Calipers	5 min
**Trigeminal** **Nerve Testing**	Trigeminal nerve function	Spicy stimuli: mustard, wasabi, capsaicin	1 min
**Forced Choice** **Taste Test**	Detect malingering	Distilled water Tastant solutions (see Table II) Medicine cups	5 min
**Three-** **Drop Taste** **Threshold Test**	Gustatory function	Distilled water Tastant solutions (see Table II) Droppers	20–30 min

## Diagnostic assessment

### Smell testing

Given that much of a patient’s subjective perception of “taste” usually involves a contribution of odor via the olfactory system ^9^ screening of olfactory function is generally performed in a taste evaluation. The extent to which this patient’s dysgeusia was influenced by smell was assessed using the Smell Identification Test (SIT; Sensonics International, Haddon Heights, N.J.). The patient scored 34/40 correct responses on this test, putting him in the 33rd percentile for age and gender, categorized as normosmia ^10^. This finding indicates that disordered smell is not a contributing factor in this patient’s dysgeusia.

### Two-point tactile discrimination

Two-point discrimination is a common neurological test of somatosensory function, and here it was utilized to assess the tongue. Calipers were used to present either one or two tactile stimuli to the tongue and the patient was asked to indicate if he felt one point or two. An ascending threshold was established by beginning with the detection points at 2 mm and gradually moving them apart until the patient perceived two distinct points. This was completed in each of four quadrants of the tongue including left posterior, right posterior, left anterior, and right anterior until the two-point detection threshold was reached. Two-point detection thresholds were as follows:

- left posterior = >23 mm,
- right posterior = 22 mm,
- left anterior = 18mm,
- right anterior = 16 mm.

These thresholds were notably elevated relative to published means of 1.09 mm in the anterior region, 2.64 mm in the canine region, and 8.08 mm in the posterior region ^11^.

### Trigeminal nerve testing

Many chemicals that stimulate taste buds also stimulate trigeminal neurons when presented at high concentrations ^12^. Conversely, compounds such as capsaicin and mustard oil elicit responses from trigeminal fibers but not from taste cells. Therefore, to test trigeminal involvement in the patient’s dysgeusia, small boluses of mustard and chili pepper sauce were presented across the dorsal surface of the tongue using a cotton tip applicator and the patient was asked to describe his perception of each. The patient was able to detect both stimuli, describing the mustard as “sour” and guessing correctly that it was mustard, and describing the chili pepper sauce as “hot” and “spicy”. These results indicate that trigeminal responses detected by nociceptive nerve fibers of the tongue were functional.

### Forced choice test

To distinguish ageusia from malingering, the patient was presented with 6 trials of a forced choice task, whereby the patient was asked to swish 10 ml of a detectable tastant concentration (the highest concentrations of sucrose and sodium chloride as outlined in Table 2) or its diluent (distilled water) and determine which contained the tastant. Sweet taste (i.e. detection of sucrose) is considered the most robust across the lifespan, and coincidentally this tastant is readily available ^13^. In 6/6 trials, the patient correctly indicated the cup containing the tastant. If the subject was truly ageusic and guessing at random, they would guess the correct cup 50% of the time, whereas scores deviating from 50 percent in either direction would indicate nonchance-level performance, and suggest that the test taker knew the correct answer but purposely selected the wrong one ^14^. This quick test can be performed six times in a row if malingering is suspected, as there is only a 1 in 64 chance that an ageusic subject would guess incorrectly six times in a row. If testing is inconclusive, it is possible to detect malingering by measuring cortical responses to tastants using evoked response potentials, though this tool is not readily available in most clinics.

**Table 2.** Tastant concentrations used for taste threshold testing. 1=mild, 2=mild-moderate, 3=moderate-strong, 4=strong. g/ml indicates grams of dry tastant per milliliter of distilled water, and M indicates molarity.

	Sweet	Salty	Sour	Bitter
	*Sucrose*	*Sodium chloride*	*Citric acid*	*Quinine HCl*
**1**	0.05 g/ml (0.15 M)	0.016 g/ml (0.27 M)	0.0125 g/ml (0.07 M)	0.0001 g/ml (0.00025 M)
**2**	0.1 g/ml (0.29 M)	0.04 g/ml (0.68 M)	0.0225 g/ml (0.12 M)	0.0002 g/ml (0.0005 M)
**3**	0.2 g/ml (0.58 M)	0.1 g/ml (1.71 M)	0.041 g/ml (0.21 M)	0.0006 g/ml (0.0015 M)
**4**	0.4 g/ml (1.17 M)	0.25 g/ml (4.28 M)	0.075 g/ml (0.39 M)	0.0015 g/ml (0.0038 M)

***Three-drop taste threshold test*.** A forced choice taste test ^15, 16^ was used to determine detection thresholds of four tastants including sucrose (sweet), sodium chloride (salty), citric acid (sour) and quinine hydrochloride (bitter), with concentrations shown in Table 2. Each trial consisted of 3 stimuli presented in a pseudorandom order at room temperature; one stimulus was the tastant and the other two were diluent (distilled water). To present the stimuli, a single drop of liquid was squeezed from a 3 ml plastic transfer pipette onto the center of the tongue dorsum, approximately 1.5 cm from the tip. The patient was asked to close his mouth following each drop and indicate which drop (the first, second, or third) had the tastant. Testing began with the highest (4) concentration of each stimulus to see if the patient could detect it and, if detected, proceeded with the lowest concentration (1) increasing in a stairstep manner until the patient could detect the stimulus. Once a concentration was detected, the next lowest concentration was trialed and if that concentration was not detected, the detectable concentration was presented in the following trial. This cycle was repeated until there were 3 consecutive detections of a given concentration. Between each trial, the patient rinsed his mouth with tap water.

The patient was unable to detect the highest concentration of sucrose, indicating that his threshold for sweet detection is greater than 0.4 g/ml (1.17 M). Salty, bitter, and sour stimuli were consistently detected at the highest (4) concentrations only. A composite score is generated based on the concentration levels detected, and in this case the composite score placed the subject in <5 ^th^ percentile compared to age- and sex-matched controls [13]. A follow up swish and spit forced-choice test comparing the lowest (1) concentrations of sour and sweet tastants to distilled water was administered to determine if whole mouth discrimination, where posterior as well as anterior taste buds can detect the tastant at its full concentration, would be different from single drop discrimination. The 3-drop test is not a true regional test of gustatory function, as a single drop of tastant spreads across the tongue and is diluted immediately upon application. The patient immediately discriminated both low concentration solutions and correctly identified the sweet stimulus as “mildly sweet” and the sour stimulus as “sour”. These findings suggest that the patient retained some partial degree of taste function, with a more severe hypogeusia in the region of the anterior tongue where taste buds may have been more exposed.

## Discussion

Taste buds in the anterior tongue are superficial receptor end organs, making them susceptible to direct chemical injury; however, reports of toxin-induced dysgeusia in the literature are rare even in surveys of specialty clinics. Smith *et al*. ^4^ described a patient with severe hypogeusia after oral contact with ammonia that resulted in a chemical burn. Workers exposed to hydrocarbons have reported subjective disturbances in taste ^17^, and taste thresholds are elevated in workers exposed to dichromate, chromic acid, and zinc chromate ^18^. In our patient, a bathroom cleaner containing a proprietary organic salt made contact with the oral mucosa. An abbreviated toxicology study was unable to determine the specific components of the cleaner, however, based on comparison to similar cleaning materials, the presumed compound was likely urea sulfate, a mildly corrosive salt that rapidly breaks down into urea and sulfuric acid.

There are multiple potential reasons for the paucity of reported cases of chemical-induced taste dysfunction cases. First, clinical assessment of taste is not as standardized a practice as testing other senses like hearing and smell is; therefore, patients may not be objectively evaluated to determine the degree of taste dysfunction until several months or years have passed since the initial exposure. This obscures identification of a particular chemical or toxin as the causative agent. Further, taste cells are continually renewed ^19^, so a superficial mucosal injury will likely resolve over time as damaged cells are replaced. Finally, several factors known to be associated with dysgeusia may confound determination of cause and effect, including various medications ^6^, comorbid conditions like oral candidiasis ^5^, diabetes ^6^, hypothyroidism ^6^, Sjogren’s syndrome ^20^, and age ^21^.

The constellation of symptoms and signs reported by the patient in this case may also be consistent with some features associated with burning mouth syndrome (BMS). BMS affects the oral mucosa, lips and/or tongue and is characterized by the sensation of burning, tingling, or numbness in the absence of visible inflammation or lesions ^22^. BMS has been associated with two factors detailed in this report: angiotensin converting enzyme (ACE) inhibitors ^22^ and oral candidiasis ^22^. ACE inhibitors inhibit zinc action in the salivary glands and taste receptor cells thereby reducing saliva production and affecting taste ^23^. Brown *et al*. ^24^ described two cases of BMS associated with ACE inhibitors that subsequently improved after changes in drug therapy. Oral candidiasis caused by *Candida albicans* is a common fungal infection and estimates indicate that anywhere from 36–60% ^25, 26^ of the population are carriers without clinical symptoms. Sakashita *et al*. ^27^, reported that 70% of *Candida* carriers in their 50’s demonstrated dysgeusia. The patient presented herein was treated for suspected (although unconfirmed) oral candidiasis approximately 6 weeks following reported onset of taste loss, suggesting that he may be a carrier for *Candida albicans*.

Thoroughly investigating the cause of a patient’s dysgeusia allows the clinician to offer potential treatment options, but it is equally important to document and determine the extent of the taste disturbance in order to track a patient’s recovery over time. Quantitative taste testing is relatively easy to perform with minimal equipment in the clinic and should be used to assess taste function of patients complaining of both acute and chronic alterations in taste. Subjective patient reports may exaggerate or minimize the nature and extent of dysgeusia, and testing can determine whether the degree of dysfunction is normal relative to age. This information can be used for patient counseling, and may also be helpful to evaluate longitudinal improvement in an objective fashion. It is also possible to detect suspected malingering by administration of simple, forced-choice tests.

Given the chronic nature of the patient’s hypogeusia, we expect that it is unlikely that he will experience spontaneous resolution at this time point. The gustatory system exhibits a robust regenerative capacity, with taste cell renewal every 10–14 days (see Barlow, 2015 ^19^ for a review). This suggests that function should have recovered within months of the incident, if the taste dysfunction was a result of superficial chemical injury to the oral cavity mucosa alone. The testing results suggest that some degree of deeper tissue injury occurred, sufficient to affect progenitor cells in the base of tongue papillae, and/or to sufficient depth such that permanent neuronal dysfunction resulted. Medical treatment options that could be attempted include discontinuing lisinopril and trialing a different (nonsulfhydryl) ACE inhibitor ^23^, or dietary supplementation with selenium methionine ^28^. Additional testing and treatment for diseases associated with taste dysfunction (e.g., diabetes, Sjorgren’s syndrome, vitamin deficiencies, and hypothyroidism) can also be performed if signs or symptoms warrant.

We employed multiple tests targeting somatosensory function, trigeminal nerve response, taste thresholds for sweet, sour, bitter, and salty, and odor detection. There are additional tests reported in the literature that can also be used for measuring dysgeusia, some of which are commercially available. These include electrogustometry ^29^ (e.g., TR-06 Rion Electrogustometer, Sensonics, Inc.), Taste Strips (Burghart), filter paper discs ^30^, taste tablets ^31^, and subjective health-related quality-of-life questionnaires. In some clinical populations including children, patients with dementia, and malingerers, objective testing with gustatory event related potentials may be required ^32^. For a standardized taste intensity testing protocol, clinicians may opt to follow the technical manual and scoring and interpretation guidelines included in the National Institutes of Health (NIH) Toolbox, as established by the National Health and Nutrition Examination Survey (NHANES) ^33^. As a very basic measure, it is also possible to create testing solutions from store-bought sugar (low concentration=3 tsp; high concentration=24 tsp) and salt (low concentration=2 tsp; high concentration=14 tsp) dissolved in 8 oz (237 ml) distilled water to perform both tongue tip and whole mouth testing.

## Conclusion

 This case report adds to the limited literature on toxin-induced hypogeusia following oral exposure. We promote the concept that taste testing is relatively easy to perform and should be completed as soon as possible following an incident in order to determine the extent of injury and track improvement in function over time.

## Consent

Written informed consent for publication of their clinical details and clinical images was obtained from the patient.
