# Supplementary material for: Case Report: Diagnosis of hypogeusia after oral exposure to commercial cleaning agent and considerations for clinical taste testing
Source: F1000Res. 2017 Jun 19;6:373. Originally published 2017 Mar 28. [Version 2] doi: 10.12688/f1000research.11241.2 (PMC5490477; doi:10.12688/f1000research.11241.2)
Supplement: Peer review history

# Peer review: Referee response for version 2

**DOI:** 10.5256/f1000research.12932.r23597

**Published:** 2017-06-26

**Authors:** Christian A. Müller, Kilian Fellinger

**Affiliations:** 1 Department of Otorhinolaryngology, Head and Neck Surgery, Medical University Vienna, Wien, Austria

Thank you for revising your Case Report. All our concerns have been addressed adequately. We have no further comments to make.

We have read this submission. We believe that we have an appropriate level of expertise to confirm that it is of an acceptable scientific standard.

# Peer review: Referee response for version 1

**DOI:** 10.5256/f1000research.12128.r22193

**Published:** 2017-05-31

**Authors:** Baslie N. Landis

**Affiliations:** 1 Department of Otorhinolaryngology, Head and Neck Surgery, Geneva University Hospitals, Medical School, University of Geneva, Geneva, Switzerland

**General Comments:**

 The authors present a case of a patient who has accidentally ingested a cleaning agent and has long lasting oral taste and sensitivity problems.

**Major Comments:**

 Although interesting, this case does not add much to the understanding of the underling mechanism.

 We do not learn what the composition of the cleaning agent was.

 The pictures are not really pathological. Tongues are like faces, they have a wide variety of individual differences which are not always pathological. The images suggest that the complaint and the neural damage can be seen. I do not think that if you would give these pictures to 5 ENT specialists without any history they would label them as pathological (unlike a clinical image where no text is needed and everybody knows the diagnosis).

 Finally, the authors mix two things that do not fit together. There is an average case report and a general overview about taste testing. I think this is a kind of not suitable mix. The overview is too short and superficial and not critically discussed. The case report is not well discussed (as the discussion is about taste tests and not about the case). There is no further lab tests done for the case to rule out other diseases (Vitamin deficiencies, Sjögren, metabolic disorders, etc..). 1.5 year follow up is short for taste disorders. Recovery of severe dysgeusia takes up to 2 years (see reference [1])

I have read this submission. I believe that I have an appropriate level of expertise to state that I do not consider it to be of an acceptable scientific standard, for reasons outlined above.

## References

[1] HeiserCLandisBNGigerRCao VanHGuinandNHörmannKStuckBA: Taste disorders after tonsillectomy: a long-term follow-up. *Laryngoscope*.2012;122(6) : 10.1002/lary.23270 1265-6 10.1002/lary.23270 22460623

# Peer review: Referee response for version 1

**DOI:** 10.5256/f1000research.12128.r22595

**Published:** 2017-05-30

**Authors:** Christian A. Müller, Kilian Fellinger

**Affiliations:** 1 Department of Otorhinolaryngology, Head and Neck Surgery, Medical University Vienna, Wien, Austria

First, we would like to thank the authors that they have illustrated the clinical importance of taste disorders and related diagnostic procedures based on this interesting case report.

 Thorough medical history and validated assessment of sensory acuity is mandatory during the management of patients with smell and taste disorders. Consequently, we would like to suggest some revisions to the case report.

 Although important causes of taste disorders are mentioned, some other causes, e.g., surgical causes, such as tonsillectomy and middle ear surgery, as well as psychogenic causes should be added (1-3).

 It would be interesting if there was a decrease in body-mass-index within the last year or if it was stable. It would also be interesting if there were any changes in medication since the accident and why and how often the patient took naproxen, as it may cause stomatitis and glossitis.

 It would be interesting if the patient reported complaints of flavor perception.

 With regard to the performance of the three-drop taste threshold, it should be mentioned that the usual way of testing starts with the lowest concentration in order to avoid adaptation. Moreover, this test measures gustatory function of the whole mouth similar to the swish and spit test.

 The authors used a two-alternative forced-choice taste test and concluded that every deviation from a 50 percent result in ageusic patients might be suspicious of malingering. However, it has to be stated that this is not always the case and that simple and quick tests cannot guarantee the suggested confidence in detection of malingering. In some circumstances more elaborate tests (e.g., event-related potentials) are needed.

 In summary, we would like to thank the authors for this interesting case report, as further research in the field of rare causes of taste disorders are necessary.

We have read this submission. We believe that we have an appropriate level of expertise to confirm that it is of an acceptable scientific standard, however we have significant reservations, as outlined above.

## References

[1] MuellerCAKhatibSLandisBNTemmelAFHummelT: Gustatory function after tonsillectomy. *Arch Otolaryngol Head Neck Surg*.2007;133(7) : 10.1001/archotol.133.7.668 668-71 10.1001/archotol.133.7.668 17638779

[2] MuellerCAKhatibSNakaATemmelAFHummelT: Clinical assessment of gustatory function before and after middle ear surgery: a prospective study with a two-year follow-up period. *Ann Otol Rhinol Laryngol*.2008;117(10) : 10.1177/000348940811701012 769-73 10.1177/000348940811701012 18998507

[3] KazourFRichaSDesmidtTLemaireMAtanasovaBEl HageW: Olfactory and gustatory functions in bipolar disorders: A systematic review. *Neurosci Biobehav Rev*.2017;80: 10.1016/j.neubiorev.2017.05.009 69-79 10.1016/j.neubiorev.2017.05.009 28506924

# Peer review: Referee response for version 1

**DOI:** 10.5256/f1000research.12128.r21955

**Published:** 2017-04-19

**Authors:** Claudio Augusto Marroni, Sabrina A. Fernandes

**Affiliations:** 1 Post Graduate Program in Medicine: Hepatology, Federal University of Health Sciences of Porto Alegre, Porto Alegre, Brazil

Firstly, we would like to congratulate the authors for the choice of the case, an interesting subject of great scientific relevance. The structuring of the case report is well documented and with a helpful review of the scientific literature on the hypotheses of the patient's clinical condition against hypogeusia.

 We have developed a line of argument on the subject, raised some issues that we consider important to contribute to the understanding of the case.

 The tongue is a muscular organ located on the floor of the mouth, its root is the posterior part, the apex lies in the anterior extremity and the dorsum is divided by a middle groove in symmetrical halves. It contributes to chewing by placing food between the teeth and plays an important role in deglutition and articulation of speech sounds ^(1)^.

 The shape of the tongue presents a characteristic appearance due to the presence of small protrusions, called lingual or gustatory papillae, that are distributed in great quantity before the terminal sulcus. The taste buds are small appendages filled with sensory cells. These cells are attached to our brain by nerve fibers ^(1)^.

 The papillae of the tongue are composed of connective tissue covered by stratified squamous epithelium and, by their appearance, are classified into four types: filiform, fungiform, goblet or circumvalate and foliaceous or foliate ^(1)^.

 The taste buds are responsible for the palate. There are names for disturbances involving the taste buds. When the sensitivity of the palate is diminished, it is classified by hypogeusia; The altered sensitivity is called dysgeusia and, finally, the ageusia is the absence of palate ^(2)^.

 Decreased palate may be associated with clinical malnutrition in diabetes mellitus, Cushing's disease, systemic arterial hypertension, obesity, adrenalectomy, neoplasias and chronic diseases, drug consumption, radiotherapy and surgical interventions, aging, saliva composition , Smoking and eating habits, mood and feelings of hunger or satiety ^(2)^.

 The reduction of the foleal papillae occurs significantly with age and the location of the dysgeusia of the evaluated patient is in the same location of these papillae.

 Dysgeusia is directly related to the characteristic of the taste buds. The taste buds present four types of cells, type I, II, III and IV, which are responsible for the transduction of the taste signal and, according to the disease that the patient may present, these cells may have a disruption.

***Type I Cells***

 Type I cells are the most abundant in the mammalian gustatory system ^(3)^ and are responsible for the hydrolysis of a portion of extracellular ATP which is a neurotransmitter of the palate as well as glutamate. Type I cells appear to be involved in the synaptic transmission that encloses and restricts signal propagation by the transmitter, performed in the central nervous system by the glial cells ^(4)^.

 In addition, type I cells have a homeostasis power through K ^+^ channels ^(2)^. During prolonged courses of action potentials stimulated by intense taste stimulation, type I cells release K ^+^ which accumulates in the interstitial limited spaces of the taste bud and decrease the excitability of the other cell types. Thus, type I cells appear to function as glial cells in taste buds ^(3)^.

 Type I cells may have ionic currents implicated in taste signal transduction(4). Although it is the most abundant type of cell in the palate, very little is known about type I cells.

***Type II Cells***

 Type II cells function within the taste buds and are incorporated into the plasma membrane of these cells, where receptors bind to bitter, sweet, or umami compounds. These flavor receptors are coupled to the GPCR-G protein (second messenger) of receptors with seven transmembrane domains ^(4)^.

 Type II cells express Na ^+^ and K ^+^ ion channels essential for the production of action potentials and structural subunits of "key" readers for ATP secretion. Any type II cell can bind to the GPCR-specific G protein family to identify taste, such as sweet or bitter, but not both ^(4)^.

 In recognition of their role as the main detectors of these palate classes, type II cells were renamed "receptor" cells. Type II cells do not appear to be directly stimulated by acid or salty ^(4)^.

 The recipient cells do not form an ultrastructure capable of performing synapses for certain flavors. Presumably, the nerve fibers of these cells have different forms of afferent synaptic connections. Signals transmitted from afferent receptor sensory cells or other cells within the gustatory papilla should be made by unconventional mechanisms, i.e. without the involvement of synaptic vesicles ^(5)^.

***Type III Cells***

 The consensus is that type III cells express proteins associated with synapses and that form synaptic junctions with nerve terminals ^(5)^. These cells express a number of genes, surface cell adhesion molecules, enzymes for the synthesis of at least two neurotransmitters and the Ca ^++^ ion channels typically associated with the release of neurotransmitters ^(5)^.

 Type III cells, which express synaptic proteins and rapid Ca ^++^-dependent depolarization, are characterized as "presynaptic." Type III cells, because they are signal receptors (presynaptic cells), are also excitable and express a complement of Na ^+^ channels and K ^+^ channels to support action potentials ^(5)^.

 Innervation where the synapse of type III cells, which appears to be by afferent connections, is not known. In addition to the above-mentioned neuronal properties, presynaptic cells also respond directly to stimuli to bitter taste and carbonate solutions and are presumably the cells responsible for signaling such sensations ^(5)^.

***Type IV Cells***

 The type IV cell is characterized by having a spherical or ovoid shape that does not extend to taste processes and is likely to be undifferentiated cell of the palate or immature ^(6)^.

 Some researchers identify type IV cells as repositories in the process of apoptosis of the others. This cell class would be the progenitor, guaranteeing the homeostasis of the other groups. Its real functions and characterization as basal cell are to be clarified ^(7,8)^.

 In the presented report we can raise the hypothesis that a deformity occurred in the structure of these cells of rapid prolifeferation, significantly compromising the signal transmission.

 We know that zinc is responsible for the formation of alpha-gustine, the protein responsible for taste, an alternative could have been offered a treatment of chelated zinc for three months, waiting for the recovery of the papillae.

 Once again, we congratulate the work and note the importance of improving the early identification of palate dysfunction in the clinical area, since numerous diseases present this clinical picture frequently and end up leading to other comorbidities.

We have read this submission. We believe that we have an appropriate level of expertise to confirm that it is of an acceptable scientific standard.

## References

[1] MurrayRG: The ultrastructure of taste buds. *In: Friedmann I. The Ultrastructure of sensory organs. North Holland: Amsterdam*.1973;1-81

[2] DavidsonHIPattisonRMRichardsonRA: Clinical undernutrition states and their influence on taste. *Proc Nutr Soc*.1998;57(4) :633-8 1009612710.1079/pns19980092

[3] JungHSAkitaKKimJY: Spacing patterns on tongue surface-gustatory papilla. *Int J Dev Biol*.2004;48(2-3) : 10.1387/ijdb.041824hj 157-61 10.1387/ijdb.041824hj 15272380

[4] FingerTEDanilovaVBarrowsJBartelDLVigersAJStoneLHellekantGKinnamonSC: ATP signaling is crucial for communication from taste buds to gustatory nerves. *Science*.2005;310(5753) : 10.1126/science.1118435 1495-9 10.1126/science.1118435 16322458

[5] VandenbeuchAKinnamonSC: Why do taste cells generate action potentials?. *J Biol*.2009;8(4) : 10.1186/jbiol138 42 10.1186/jbiol138 19439032PMC2688909

[6] SimonSAde AraujoIEGutierrezRNicolelisMA: The neural mechanisms of gustation: a distributed processing code. *Nat Rev Neurosci*.2006;7(11) : 10.1038/nrn2006 890-901 10.1038/nrn2006 17053812

[7] GaoNLuMEcheverriFLaitaBKalabatDWilliamsMEHeveziPZlotnikAMoyerBD: Voltage-gated sodium channels in taste bud cells. *BMC Neurosci*.2009;10: 10.1186/1471-2202-10-20 20 10.1186/1471-2202-10-20 19284629PMC2660338

[8] ChaudhariNRoperSD: The cell biology of taste. *J Cell Biol*.2010;190(3) : 10.1083/jcb.201003144 285-96 10.1083/jcb.201003144 20696704PMC2922655
